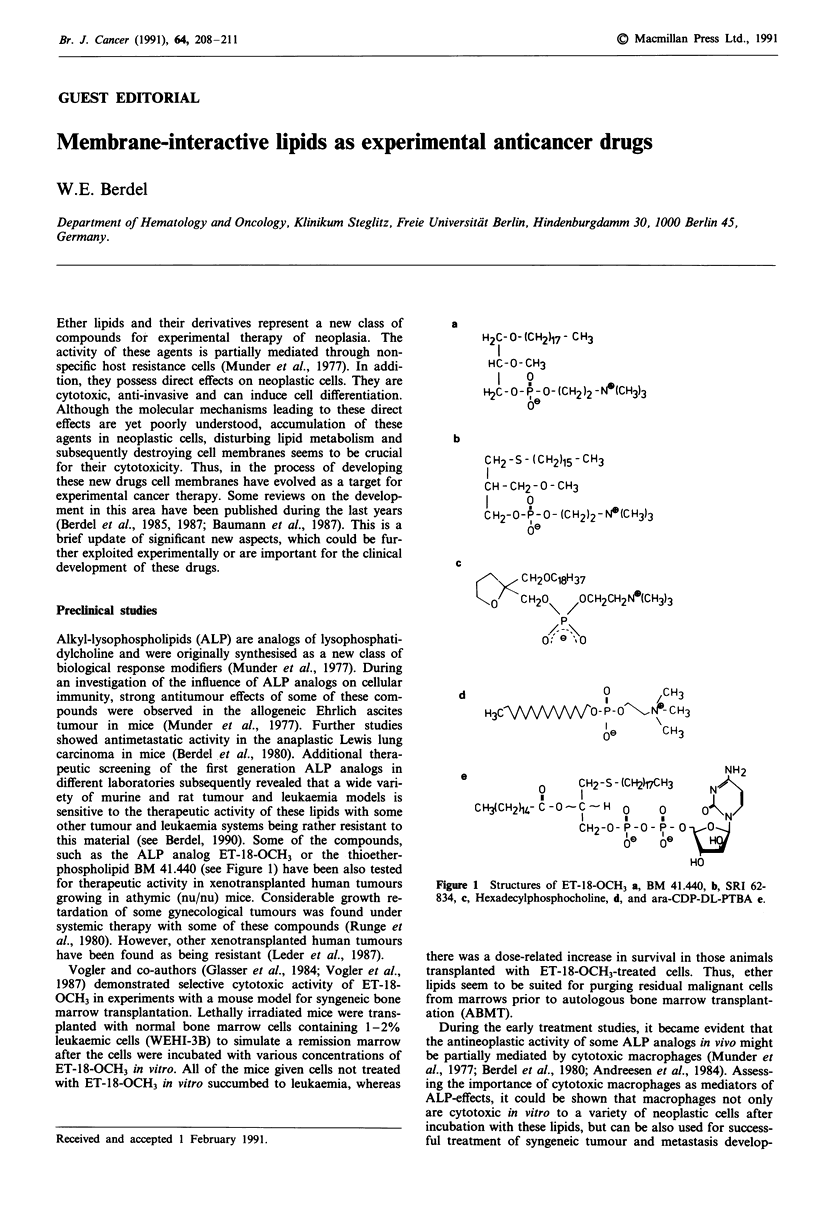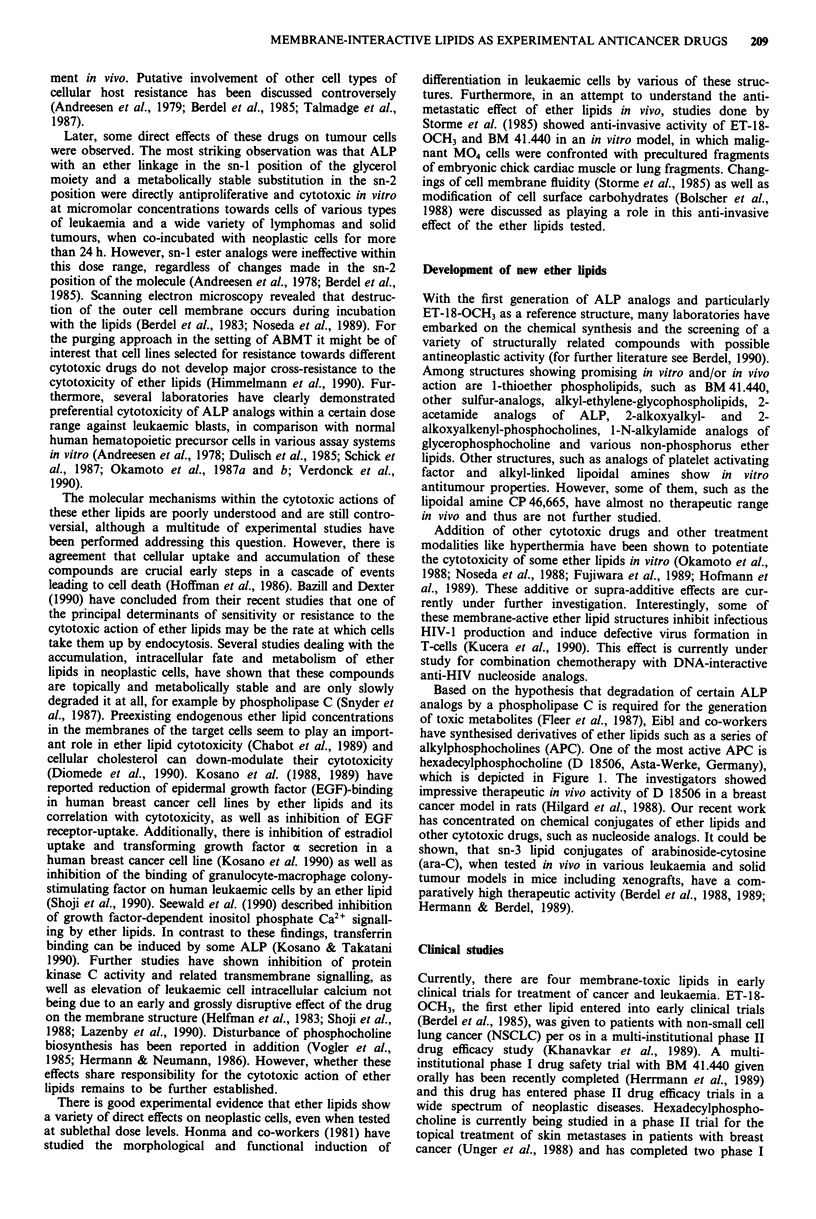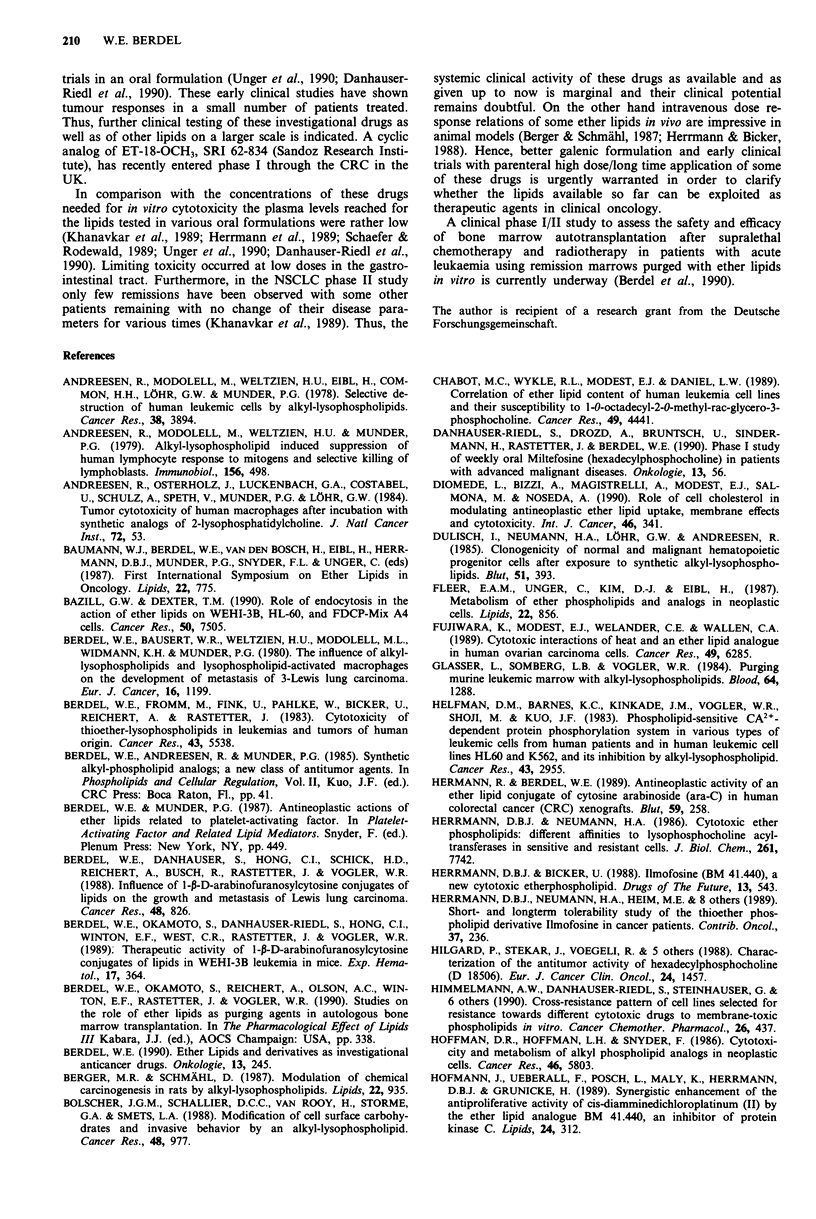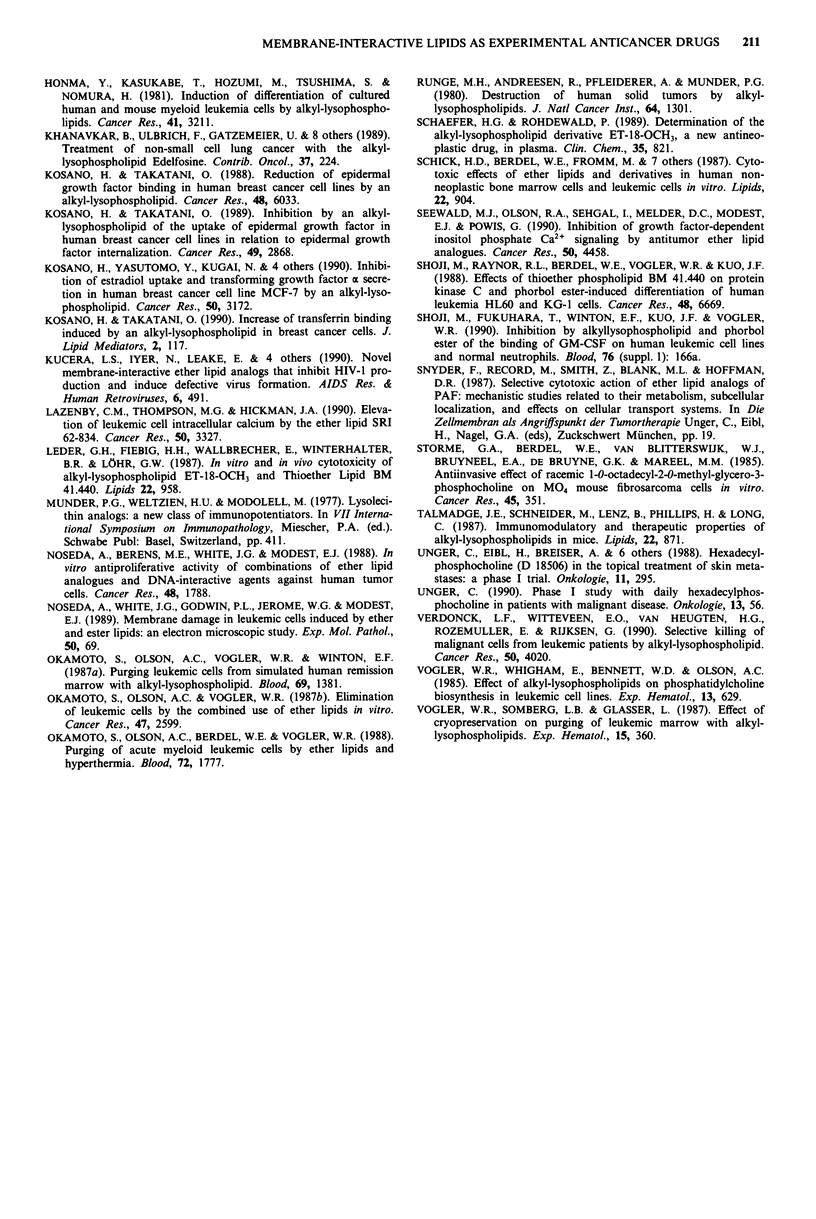# Membrane-interactive lipids as experimental anticancer drugs.

**DOI:** 10.1038/bjc.1991.277

**Published:** 1991-08

**Authors:** W. E. Berdel


Br. J. Cancer (1991), 64, 208–211 © Macmillan Press Ltd., 1991

GUEST EDITORIAL

### Membrane-interactive lipids as experimental anticancer drugs

W.E. Berdel

*Department of Hematology and Oncology, Klinikum Steglitz, Freie Universität Berlin, Hindenburgdamm 30, 1000 Berlin 45, Germany.*

Ether lipids and their derivatives represent a new class of compounds for experimental therapy of neoplasia. The activity of these agents is partially mediated through non-specific host resistance cells (Munder *et al.*, 1977). In addition, they possess direct effects on neoplastic cells. They are cytotoxic, anti-invasive and can induce cell differentiation. Although the molecular mechanisms leading to these direct effects are yet poorly understood, accumulation of these agents in neoplastic cells, disturbing lipid metabolism and subsequently destroying cell membranes seems to be crucial for their cytotoxicity. Thus, in the process of developing these new drugs cell membranes have evolved as a target for experimental cancer therapy. Some reviews on the development in this area have been published during the last years (Berdel *et al.*, 1985, 1987; Baumann *et al.*, 1987). This is a brief update of significant new aspects, which could be further exploited experimentally or are important for the clinical development of these drugs.

### Preclinical studies

Alkyl-lysophospholipids (ALP) are analogs of lysophosphatidylcholine and were originally synthesised as a new class of biological response modifiers (Munder *et al.*, 1977). During an investigation of the influence of ALP analogs on cellular immunity, strong antitumour effects of some of these compounds were observed in the allogeneic Ehrlich ascites tumour in mice (Munder *et al.*, 1977). Further studies showed antimetastatic activity in the anaplastic Lewis lung carcinoma in mice (Berdel *et al.*, 1980). Additional therapeutic screening of the first generation ALP analogs in different laboratories subsequently revealed that a wide variety of murine and rat tumour and leukaemia models is sensitive to the therapeutic activity of these lipids with some other tumour and leukaemia systems being rather resistant to this material (see Berdel, 1990). Some of the compounds, such as the ALP analog ET-18-$OCH_3$ or the thioether-phospholipid BM 41.440 (see Figure 1) have been also tested for therapeutic activity in xenotransplanted human tumours growing in athymic (nu/nu) mice. Considerable growth retardation of some gynecological tumours was found under systemic therapy with some of these compounds (Runge *et al.*, 1980). However, other xenotransplanted human tumours have been found as being resistant (Leder *et al.*, 1987).

Vogler and co-authors (Glasser *et al.*, 1984; Vogler *et al.*, 1987) demonstrated selective cytotoxic activity of ET-18-$OCH_3$ in experiments with a mouse model for syngeneic bone marrow transplantation. Lethally irradiated mice were transplanted with normal bone marrow cells containing 1–2% leukaemic cells (WEHI-3B) to simulate a remission marrow after the cells were incubated with various concentrations of ET-18-$OCH_3$ *in vitro*. All of the mice given cells not treated with ET-18-$OCH_3$ *in vitro* succumbed to leukaemia, whereas

**Figure 1** Structures of ET-18-$OCH_3$ **a**, BM 41.440, **b**, SRI 62-834, **c**, Hexadecylphosphocholine, **d**, and ara-CDP-DL-PTBA **e**.

there was a dose-related increase in survival in those animals transplanted with ET-18-$OCH_3$-treated cells. Thus, ether lipids seem to be suited for purging residual malignant cells from marrows prior to autologous bone marrow transplantation (ABMT).

During the early treatment studies, it became evident that the antineoplastic activity of some ALP analogs *in vivo* might be partially mediated by cytotoxic macrophages (Munder *et al.*, 1977; Berdel *et al.*, 1980; Andreesen *et al.*, 1984). Assessing the importance of cytotoxic macrophages as mediators of ALP-effects, it could be shown that macrophages not only are cytotoxic *in vitro* to a variety of neoplastic cells after incubation with these lipids, but can be also used for successful treatment of syngeneic tumour and metastasis develop-

Received and accepted 1 February 1991.

ment *in vivo*. Putative involvement of other cell types of cellular host resistance has been discussed controversely (Andreesen *et al.*, 1979; Berdel *et al.*, 1985; Talmadge *et al.*, 1987).

Later, some direct effects of these drugs on tumour cells were observed. The most striking observation was that ALP with an ether linkage in the sn-1 position of the glycerol moiety and a metabolically stable substitution in the sn-2 position were directly antiproliferative and cytotoxic *in vitro* at micromolar concentrations towards cells of various types of leukaemia and a wide variety of lymphomas and solid tumours, when co-incubated with neoplastic cells for more than 24 h. However, sn-1 ester analogs were ineffective within this dose range, regardless of changes made in the sn-2 position of the molecule (Andreesen *et al.*, 1978; Berdel *et al.*, 1985). Scanning electron microscopy revealed that destruction of the outer cell membrane occurs during incubation with the lipids (Berdel *et al.*, 1983; Noseda *et al.*, 1989). For the purging approach in the setting of ABMT it might be of interest that cell lines selected for resistance towards different cytotoxic drugs do not develop major cross-resistance to the cytotoxicity of ether lipids (Himmelmann *et al.*, 1990). Furthermore, several laboratories have clearly demonstrated preferential cytotoxicity of ALP analogs within a certain dose range against leukaemic blasts, in comparison with normal human hematopoietic precursor cells in various assay systems *in vitro* (Andreesen *et al.*, 1978; Dulisch *et al.*, 1985; Schick *et al.*, 1987; Okamoto *et al.*, 1987*a* and *b*; Verdonck *et al.*, 1990).

The molecular mechanisms within the cytotoxic actions of these ether lipids are poorly understood and are still controversial, although a multitude of experimental studies have been performed addressing this question. However, there is agreement that cellular uptake and accumulation of these compounds are crucial early steps in a cascade of events leading to cell death (Hoffman *et al.*, 1986). Bazill and Dexter (1990) have concluded from their recent studies that one of the principal determinants of sensitivity or resistance to the cytotoxic action of ether lipids may be the rate at which cells take them up by endocytosis. Several studies dealing with the accumulation, intracellular fate and metabolism of ether lipids in neoplastic cells, have shown that these compounds are topically and metabolically stable and are only slowly degraded it at all, for example by phospholipase C (Snyder *et al.*, 1987). Preexisting endogenous ether lipid concentrations in the membranes of the target cells seem to play an important role in ether lipid cytotoxicity (Chabot *et al.*, 1989) and cellular cholesterol can down-modulate their cytotoxicity (Diomede *et al.*, 1990). Kosano *et al.* (1988, 1989) have reported reduction of epidermal growth factor (EGF)-binding in human breast cancer cell lines by ether lipids and its correlation with cytotoxicity, as well as inhibition of EGF receptor-uptake. Additionally, there is inhibition of estradiol uptake and transforming growth factor α secretion in a human breast cancer cell line (Kosano *et al.* 1990) as well as inhibition of the binding of granulocyte-macrophage colony-stimulating factor on human leukaemic cells by an ether lipid (Shoji *et al.*, 1990). Seewald *et al.* (1990) described inhibition of growth factor-dependent inositol phosphate $Ca^{2+}$ signalling by ether lipids. In contrast to these findings, transferrin binding can be induced by some ALP (Kosano & Takatani 1990). Further studies have shown inhibition of protein kinase C activity and related transmembrane signalling, as well as elevation of leukaemic cell intracellular calcium not being due to an early and grossly disruptive effect of the drug on the membrane structure (Helfman *et al.*, 1983; Shoji *et al.*, 1988; Lazenby *et al.*, 1990). Disturbance of phosphocholine biosynthesis has been reported in addition (Vogler *et al.*, 1985; Hermann & Neumann, 1986). However, whether these effects share responsibility for the cytotoxic action of ether lipids remains to be further established.

There is good experimental evidence that ether lipids show a variety of direct effects on neoplastic cells, even when tested at sublethal dose levels. Honma and co-workers (1981) have studied the morphological and functional induction of differentiation in leukaemic cells by various of these structures. Furthermore, in an attempt to understand the anti-metastatic effect of ether lipids *in vivo*, studies done by Storme *et al.* (1985) showed anti-invasive activity of ET-18-$OCH_3$ and BM 41.440 in an *in vitro* model, in which malignant $MO_4$ cells were confronted with precultured fragments of embryonic chick cardiac muscle or lung fragments. Changings of cell membrane fluidity (Storme *et al.*, 1985) as well as modification of cell surface carbohydrates (Bolscher *et al.*, 1988) were discussed as playing a role in this anti-invasive effect of the ether lipids tested.

### Development of new ether lipids

With the first generation of ALP analogs and particularly ET-18-$OCH_3$ as a reference structure, many laboratories have embarked on the chemical synthesis and the screening of a variety of structurally related compounds with possible antineoplastic activity (for further literature see Berdel, 1990). Among structures showing promising *in vitro* and/or *in vivo* action are 1-thioether phospholipids, such as BM 41.440, other sulfur-analogs, alkyl-ethylene-glycophospholipids, 2-acetamide analogs of ALP, 2-alkoxyalkyl- and 2-alkoxyalkenyl-phosphocholines, 1-N-alkylamide analogs of glycerophosphocholine and various non-phosphorus ether lipids. Other structures, such as analogs of platelet activating factor and alkyl-linked lipoidal amines show *in vitro* antitumour properties. However, some of them, such as the lipoidal amine CP 46,665, have almost no therapeutic range *in vivo* and thus are not further studied.

Addition of other cytotoxic drugs and other treatment modalities like hyperthermia have been shown to potentiate the cytotoxicity of some ether lipids *in vitro* (Okamoto *et al.*, 1988; Noseda *et al.*, 1988; Fujiwara *et al.*, 1989; Hofmann *et al.*, 1989). These additive or supra-additive effects are currently under further investigation. Interestingly, some of these membrane-active ether lipid structures inhibit infectious HIV-1 production and induce defective virus formation in T-cells (Kucera *et al.*, 1990). This effect is currently under study for combination chemotherapy with DNA-interactive anti-HIV nucleoside analogs.

Based on the hypothesis that degradation of certain ALP analogs by a phospholipase C is required for the generation of toxic metabolites (Fleer *et al.*, 1987), Eibl and co-workers have synthesised derivatives of ether lipids such as a series of alkylphosphocholines (APC). One of the most active APC is hexadecylphosphocholine (D 18506, Asta-Werke, Germany), which is depicted in Figure 1. The investigators showed impressive therapeutic *in vivo* activity of D 18506 in a breast cancer model in rats (Hilgard *et al.*, 1988). Our recent work has concentrated on chemical conjugates of ether lipids and other cytotoxic drugs, such as nucleoside analogs. It could be shown, that sn-3 lipid conjugates of arabinoside-cytosine (ara-C), when tested *in vivo* in various leukaemia and solid tumour models in mice including xenografts, have a comparatively high therapeutic activity (Berdel *et al.*, 1988, 1989; Hermann & Berdel, 1989).

### Clinical studies

Currently, there are four membrane-toxic lipids in early clinical trials for treatment of cancer and leukaemia. ET-18-$OCH_3$, the first ether lipid entered into early clinical trials (Berdel *et al.*, 1985), was given to patients with non-small cell lung cancer (NSCLC) per os in a multi-institutional phase II drug efficacy study (Khanavkar *et al.*, 1989). A multi-institutional phase I drug safety trial with BM 41.440 given orally has been recently completed (Herrmann *et al.*, 1989) and this drug has entered phase II drug efficacy trials in a wide spectrum of neoplastic diseases. Hexadecylphosphocholine is currently being studied in a phase II trial for the topical treatment of skin metastases in patients with breast cancer (Unger *et al.*, 1988) and has completed two phase I

trials in an oral formulation (Unger *et al.*, 1990; Danhauser-Riedl *et al.*, 1990). These early clinical studies have shown tumour responses in a small number of patients treated. Thus, further clinical testing of these investigational drugs as well as of other lipids on a larger scale is indicated. A cyclic analog of ET-18-$OCH_3$, SRI 62-834 (Sandoz Research Institute), has recently entered phase I through the CRC in the UK.

In comparison with the concentrations of these drugs needed for *in vitro* cytotoxicity the plasma levels reached for the lipids tested in various oral formulations were rather low (Khanavkar *et al.*, 1989; Herrmann *et al.*, 1989; Schaefer & Rodewald, 1989; Unger *et al.*, 1990; Danhauser-Riedl *et al.*, 1990). Limiting toxicity occurred at low doses in the gastrointestinal tract. Furthermore, in the NSCLC phase II study only few remissions have been observed with some other patients remaining with no change of their disease parameters for various times (Khanavkar *et al.*, 1989). Thus, the systemic clinical activity of these drugs as available and as given up to now is marginal and their clinical potential remains doubtful. On the other hand intravenous dose response relations of some ether lipids *in vivo* are impressive in animal models (Berger & Schmähl, 1987; Herrmann & Bicker, 1988). Hence, better galenic formulation and early clinical trials with parenteral high dose/long time application of some of these drugs is urgently warranted in order to clarify whether the lipids available so far can be exploited as therapeutic agents in clinical oncology.

A clinical phase I/II study to assess the safety and efficacy of bone marrow autotransplantation after supralethal chemotherapy and radiotherapy in patients with acute leukaemia using remission marrows purged with ether lipids *in vitro* is currently underway (Berdel *et al.*, 1990).

The author is recipient of a research grant from the Deutsche Forschungsgemeinschaft.

### References

ANDREESEN, R., MODOLELL, M., WELTZIEN, H.U., EIBL, H., COMMON, H.H., LÖHR, G.W. & MUNDER, P.G. (1978). Selective destruction of human leukemic cells by alkyl-lysophospholipids. *Cancer Res.*, **38**, 3894.

ANDREESEN, R., MODOLELL, M., WELTZIEN, H.U. & MUNDER, P.G. (1979). Alkyl-lysophospholipid induced suppression of human lymphocyte response to mitogens and selective killing of lymphoblasts. *Immunobiol.*, **156**, 498.

ANDREESEN, R., OSTERHOLZ, J., LUCKENBACH, G.A., COSTABEL, U., SCHULZ, A., SPETH, V., MUNDER, P.G. & LÖHR, G.W. (1984). Tumor cytotoxicity of human macrophages after incubation with synthetic analogs of 2-lysophosphatidylcholine. *J. Natl Cancer Inst.*, **72**, 53.

BAUMANN, W.J., BERDEL, W.E., VAN DEN BOSCH, H., EIBL, H., HERRMANN, D.B.J., MUNDER, P.G., SNYDER, F.L. & UNGER, C. (eds) (1987). First International Symposium on Ether Lipids in Oncology. *Lipids*, **22**, 775.

BAZILL, G.W. & DEXTER, T.M. (1990). Role of endocytosis in the action of ether lipids on WEHI-3B, HL-60, and FDCP-Mix A4 cells. *Cancer Res.*, **50**, 7505.

BERDEL, W.E., BAUSERT, W.R., WELTZIEN, H.U., MODOLELL, M.L., WIDMANN, K.H. & MUNDER, P.G. (1980). The influence of alkyl-lysophospholipids and lysophospholipid-activated macrophages on the development of metastasis of 3-Lewis lung carcinoma. *Eur. J. Cancer*, **16**, 1199.

BERDEL, W.E., FROMM, M., FINK, U., PAHLKE, W., BICKER, U., REICHERT, A. & RASTETTER, J. (1983). Cytotoxicity of thioether-lysophospholipids in leukemias and tumors of human origin. *Cancer Res.*, **43**, 5538.

BERDEL, W.E., ANDREESEN, R. & MUNDER, P.G. (1985). Synthetic alkyl-phospholipid analogs; a new class of antitumor agents. In *Phospholipids and Cellular Regulation*, Vol. II, Kuo, J.F. (ed.). CRC Press: Boca Raton, Fl., pp. 41.

BERDEL, W.E. & MUNDER, P.G. (1987). Antineoplastic actions of ether lipids related to platelet-activating factor. In *Platelet-Activating Factor and Related Lipid Mediators*. Snyder, F. (ed.). Plenum Press: New York, NY, pp. 449.

BERDEL, W.E., DANHAUSER, S., HONG, C.I., SCHICK, H.D., REICHERT, A., BUSCH, R., RASTETTER, J. & VOGLER, W.R. (1988). Influence of 1-β-D-arabinofuranosylcytosine conjugates of lipids on the growth and metastasis of Lewis lung carcinoma. *Cancer Res.*, **48**, 826.

BERDEL, W.E., OKAMOTO, S., DANHAUSER-RIEDL, S., HONG, C.I., WINTON, E.F., WEST, C.R., RASTETTER, J. & VOGLER, W.R. (1989). Therapeutic activity of 1-β-D-arabinofuranosylcytosine conjugates of lipids in WEHI-3B leukemia in mice. *Exp. Hematol.*, **17**, 364.

BERDEL, W.E., OKAMOTO, S., REICHERT, A., OLSON, A.C., WINTON, E.F., RASTETTER, J. & VOGLER, W.R. (1990). Studies on the role of ether lipids as purging agents in autologous bone marrow transplantation. In *The Pharmacological Effect of Lipids III* Kabara, J.J. (ed.), AOCS Champaign: USA, pp. 338.

BERDEL, W.E. (1990). Ether Lipids and derivatives as investigational anticancer drugs. *Onkologie*, **13**, 245.

BERGER, M.R. & SCHMÄHL, D. (1987). Modulation of chemical carcinogenesis in rats by alkyl-lysophospholipids. *Lipids*, **22**, 935.

BOLSCHER, J.G.M., SCHALLIER, D.C.C., VAN ROOY, H., STORME, G.A. & SMETS, L.A. (1988). Modification of cell surface carbohydrates and invasive behavior by an alkyl-lysophospholipid. *Cancer Res.*, **48**, 977.

CHABOT, M.C., WYKLE, R.L., MODEST, E.J. & DANIEL, L.W. (1989). Correlation of ether lipid content of human leukemia cell lines and their susceptibility to 1-0-octadecyl-2-0-methyl-rac-glycero-3-phosphocholine. *Cancer Res.*, **49**, 4441.

DANHAUSER-RIEDL, S., DROZD, A., BRUNTSCH, U., SINDERMANN, H., RASTETTER, J. & BERDEL, W.E. (1990). Phase I study of weekly oral Miltefosine (hexadecylphosphocholine) in patients with advanced malignant diseases. *Onkologie*, **13**, 56.

DIOMEDE, L., BIZZI, A., MAGISTRELLI, A., MODEST, E.J., SALMONA, M. & NOSEDA, A. (1990). Role of cell cholesterol in modulating antineoplastic ether lipid uptake, membrane effects and cytotoxicity. *Int. J. Cancer*, **46**, 341.

DULISCH, I., NEUMANN, H.A., LÖHR, G.W. & ANDREESEN, R. (1985). Clonogenicity of normal and malignant hematopoietic progenitor cells after exposure to synthetic alkyl-lysophospholipids. *Blut*, **51**, 393.

FLEER, E.A.M., UNGER, C., KIM, D.-J. & EIBL, H., (1987). Metabolism of ether phospholipids and analogs in neoplastic cells. *Lipids*, **22**, 856.

FUJIWARA, K., MODEST, E.J., WELANDER, C.E. & WALLEN, C.A. (1989). Cytotoxic interactions of heat and an ether lipid analogue in human ovarian carcinoma cells. *Cancer Res.*, **49**, 6285.

GLASSER, L., SOMBERG, L.B. & VOGLER, W.R. (1984). Purging murine leukemic marrow with alkyl-lysophospholipids. *Blood*, **64**, 1288.

HELFMAN, D.M., BARNES, K.C., KINKADE, J.M., VOGLER, W.R., SHOJI, M. & KUO, J.F. (1983). Phospholipid-sensitive $CA^{2+}$-dependent protein phosphorylation system in various types of leukemic cells from human patients and in human leukemic cell lines HL60 and K562, and its inhibition by alkyl-lysophospholipid. *Cancer Res.*, **43**, 2955.

HERMANN, R. & BERDEL, W.E. (1989). Antineoplastic activity of an ether lipid conjugate of cytosine arabinoside (ara-C) in human colorectal cancer (CRC) xenografts. *Blut*, **59**, 258.

HERRMANN, D.B.J. & NEUMANN, H.A. (1986). Cytotoxic ether phospholipids: different affinities to lysophosphocholine acyltransferases in sensitive and resistant cells. *J. Biol. Chem.*, **261**, 7742.

HERRMANN, D.B.J. & BICKER, U. (1988). Ilmofosine (BM 41.440), a new cytotoxic etherphospholipid. *Drugs of The Future*, **13**, 543.

HERRMANN, D.B.J., NEUMANN, H.A., HEIM, M.E. & 8 others (1989). Short- and longterm tolerability study of the thioether phospholipid derivative Ilmofosine in cancer patients. *Contrib. Oncol.*, **37**, 236.

HILGARD, P., STEKAR, J., VOEGELI, R. & 5 others (1988). Characterization of the antitumor activity of hexadecylphosphocholine (D 18506). *Eur. J. Cancer Clin. Oncol.*, **24**, 1457.

HIMMELMANN, A.W., DANHAUSER-RIEDL, S., STEINHAUSER, G. & 6 others (1990). Cross-resistance pattern of cell lines selected for resistance towards different cytotoxic drugs to membrane-toxic phospholipids *in vitro*. *Cancer Chemother. Pharmacol.*, **26**, 437.

HOFFMAN, D.R., HOFFMAN, L.H. & SNYDER, F. (1986). Cytotoxicity and metabolism of alkyl phospholipid analogs in neoplastic cells. *Cancer Res.*, **46**, 5803.

HOFMANN, J., UEBERALL, F., POSCH, L., MALY, K., HERRMANN, D.B.J. & GRUNICKE, H. (1989). Synergistic enhancement of the antiproliferative activity of cis-diamminedichloroplatinum (II) by the ether lipid analogue BM 41.440, an inhibitor of protein kinase C. *Lipids*, **24**, 312.

HONMA, Y., KASUKABE, T., HOZUMI, M., TSUSHIMA, S. & NOMURA, H. (1981). Induction of differentiation of cultured human and mouse myeloid leukemia cells by alkyl-lysophospholipids. *Cancer Res.*, **41**, 3211.

KHANAVKAR, B., ULBRICH, F., GATZEMEIER, U. & 8 others (1989). Treatment of non-small cell lung cancer with the alkyl-lysophospholipid Edelfosine. *Contrib. Oncol.*, **37**, 224.

KOSANO, H. & TAKATANI, O. (1988). Reduction of epidermal growth factor binding in human breast cancer cell lines by an alkyl-lysophospholipid. *Cancer Res.*, **48**, 6033.

KOSANO, H. & TAKATANI, O. (1989). Inhibition by an alkyl-lysophospholipid of the uptake of epidermal growth factor in human breast cancer cell lines in relation to epidermal growth factor internalization. *Cancer Res.*, **49**, 2868.

KOSANO, H., YASUTOMO, Y., KUGAI, N. & 4 others (1990). Inhibition of estradiol uptake and transforming growth factor α secretion in human breast cancer cell line MCF-7 by an alkyl-lysophospholipid. *Cancer Res.*, **50**, 3172.

KOSANO, H. & TAKATANI, O. (1990). Increase of transferrin binding induced by an alkyl-lysophospholipid in breast cancer cells. *J. Lipid Mediators*, **2**, 117.

KUCERA, L.S., IYER, N., LEAKE, E. & 4 others (1990). Novel membrane-interactive ether lipid analogs that inhibit HIV-1 production and induce defective virus formation. *AIDS Res. & Human Retroviruses*, **6**, 491.

LAZENBY, C.M., THOMPSON, M.G. & HICKMAN, J.A. (1990). Elevation of leukemic cell intracellular calcium by the ether lipid SRI 62-834. *Cancer Res.*, **50**, 3327.

LEDER, G.H., FIEBIG, H.H., WALLBRECHER, E., WINTERHALTER, B.R. & LÖHR, G.W. (1987). *In vitro* and *in vivo* cytotoxicity of alkyl-lysophospholipid ET-18-$OCH_3$ and Thioether Lipid BM 41.440. *Lipids* **22**, 958.

MUNDER, P.G., WELTZIEN, H.U. & MODOLELL, M. (1977). Lysolecithin analogs: a new class of immunopotentiators. In *VII International Symposium on Immunopathology*, Miescher, P.A. (ed.). Schwabe Publ: Basel, Switzerland, pp. 411.

NOSEDA, A., BERENS, M.E., WHITE, J.G. & MODEST, E.J. (1988). *In vitro* antiproliferative activity of combinations of ether lipid analogues and DNA-interactive agents against human tumor cells. *Cancer Res.*, **48**, 1788.

NOSEDA, A., WHITE, J.G., GODWIN, P.L., JEROME, W.G. & MODEST, E.J. (1989). Membrane damage in leukemic cells induced by ether and ester lipids: an electron microscopic study. *Exp. Mol. Pathol.*, **50**, 69.

OKAMOTO, S., OLSON, A.C., VOGLER, W.R. & WINTON, E.F. (1987*a*). Purging leukemic cells from simulated human remission marrow with alkyl-lysophospholipid. *Blood*, **69**, 1381.

OKAMOTO, S., OLSON, A.C. & VOGLER, W.R. (1987*b*). Elimination of leukemic cells by the combined use of ether lipids *in vitro*. *Cancer Res.*, **47**, 2599.

OKAMOTO, S., OLSON, A.C., BERDEL, W.E. & VOGLER, W.R. (1988). Purging of acute myeloid leukemic cells by ether lipids and hyperthermia. *Blood*, **72**, 1777.

RUNGE, M.H., ANDREESEN, R., PFLEIDERER, A. & MUNDER, P.G. (1980). Destruction of human solid tumors by alkyl-lysophospholipids. *J. Natl Cancer Inst.*, **64**, 1301.

SCHAEFER, H.G. & ROHDEWALD, P. (1989). Determination of the alkyl-lysophospholipid derivative ET-18-$OCH_3$, a new antineoplastic drug, in plasma. *Clin. Chem.*, **35**, 821.

SCHICK, H.D., BERDEL, W.E., FROMM, M. & 7 others (1987). Cytotoxic effects of ether lipids and derivatives in human non-neoplastic bone marrow cells and leukemic cells *in vitro*. *Lipids*, **22**, 904.

SEEWALD, M.J., OLSON, R.A., SEHGAL, I., MELDER, D.C., MODEST, E.J. & POWIS, G. (1990). Inhibition of growth factor-dependent inositol phosphate $Ca^{2+}$ signaling by antitumor ether lipid analogues. *Cancer Res.*, **50**, 4458.

SHOJI, M., RAYNOR, R.L., BERDEL, W.E., VOGLER, W.R. & KUO, J.F. (1988). Effects of thioether phospholipid BM 41.440 on protein kinase C and phorbol ester-induced differentiation of human leukemia HL60 and KG-1 cells. *Cancer Res.*, **48**, 6669.

SHOJI, M., FUKUHARA, T., WINTON, E.F., KUO, J.F. & VOGLER, W.R. (1990). Inhibition by alkyllysophospholipid and phorbol ester of the binding of GM-CSF on human leukemic cell lines and normal neutrophils. *Blood*, **76** (suppl. 1): 166a.

SNYDER, F., RECORD, M., SMITH, Z., BLANK, M.L. & HOFFMAN, D.R. (1987). Selective cytotoxic action of ether lipid analogs of PAF: mechanistic studies related to their metabolism, subcellular localization, and effects on cellular transport systems. In *Die Zellmembran als Angriffspunkt der Tumortherapie* Unger, C., Eibl, H., Nagel, G.A. (eds), Zuckschwert München, pp. 19.

STORME, G.A., BERDEL, W.E., VAN BLITTERSWIJK, W.J., BRUYNEEL, E.A., DE BRUYNE, G.K. & MAREEL, M.M. (1985). Antiinvasive effect of racemic 1-*0*-octadecyl-2-*0*-methyl-glycero-3-phosphocholine on $MO_4$ mouse fibrosarcoma cells *in vitro*. *Cancer Res.*, **45**, 351.

TALMADGE, J.E., SCHNEIDER, M., LENZ, B., PHILLIPS, H. & LONG, C. (1987). Immunomodulatory and therapeutic properties of alkyl-lysophospholipids in mice. *Lipids*, **22**, 871.

UNGER, C., EIBL, H., BREISER, A. & 6 others (1988). Hexadecylphosphocholine (D 18506) in the topical treatment of skin metastases: a phase I trial. *Onkologie*, **11**, 295.

UNGER, C. (1990). Phase I study with daily hexadecylphosphocholine in patients with malignant disease. *Onkologie*, **13**, 56.

VERDONCK, L.F., WITTEVEEN, E.O., VAN HEUGTEN, H.G., ROZEMULLER, E. & RIJKSEN, G. (1990). Selective killing of malignant cells from leukemic patients by alkyl-lysophospholipid. *Cancer Res.*, **50**, 4020.

VOGLER, W.R., WHIGHAM, E., BENNETT, W.D. & OLSON, A.C. (1985). Effect of alkyl-lysophospholipids on phosphatidylcholine biosynthesis in leukemic cell lines. *Exp. Hematol.*, **13**, 629.

VOGLER, W.R., SOMBERG, L.B. & GLASSER, L. (1987). Effect of cryopreservation on purging of leukemic marrow with alkyl-lysophospholipids. *Exp. Hematol.*, **15**, 360.